# Cognate Antigen Stimulation Generates Potent CD8^+^ Inflammatory Effector T Cells

**DOI:** 10.3389/fimmu.2013.00452

**Published:** 2013-12-16

**Authors:** Hsueh-Cheng Sung, Sara Lemos, Patricia Ribeiro-Santos, Kateryna Kozyrytska, Florence Vasseur, Agnès Legrand, Alain Charbit, Benedita Rocha, César Evaristo

**Affiliations:** ^1^Faculté de Médecine, U1020, Université Paris-Descartes, INSERM, Paris, France; ^2^Faculté de Médecine, U1002, Université Paris-Descartes, INSERM, Paris, France

**Keywords:** CD8 T cells, immune responses, effector functions, lymphocyte trapping, lymph-node shut-down-phase

## Abstract

Inflammatory reactions are believed to be triggered by innate signals and have a major protective role by recruiting innate immunity cells, favoring lymphocyte activation and differentiation, and thus contributing to the sequestration and elimination of the injurious stimuli. Although certain lymphocyte types such as TH17 cells co-participate in inflammatory reactions, their generation from the naïve pool requires the pre-existence of an inflammatory milieu. In this context, inflammation is always regarded as beginning with an innate response that may be eventually perpetuated and amplified by certain lymphocyte types. In contrast, we here show that even in sterile immunizations or in MyD88-deficient mice, CD8 T cells produce a burst of pro-inflammatory cytokines and chemokines. These functions follow opposite rules to the classic CD8 effector functions since they are generated prior to cell expansion and decline before antigen elimination. As few as 56 CD8^+^ inflammatory effector cells in a lymph node can mobilize 10^7^ cells in 24 h, including lymphocytes, natural killer cells, and several accessory cell types involved in inflammatory reactions. Thus, although inflammation modulates cognate responses, CD8 cognate responses also initiate local inflammatory reactions.

## Introduction

The main CD8 effector functions are believed to be the production of IFN-γ and cytotoxic activity (CTL), which are induced after extensive division. However, while studying CD8 T cell responses at day 4 after priming we found that these cells had other properties. We named them “inflammatory effectors” because they expressed abundant *Tgfb*, but none co-expressed *TgfbR1* and *R2*, indicating that TGF-β could only act in *trans*, where it is pro-inflammatory [reviewed in Ref. (1)]. While testing their CTL activity (by co-injecting them with antigen-loaded and non-loaded targets directly into the spleen), these effectors did not kill loaded targets, but rather induced the local retention of both antigen-loaded and non-loaded targets (2), mimicking the events described in non-specific phase of lymphocyte trapping (3–5). Indeed, during the first 2–4 days of an immune response all antigen-specific-cells dispersed throughout the body are retained in the restricted site where the antigen is first presented (a phenomenon named lymphocyte trapping). Recent studies suggested that this local retention was due to the formation of stable interactions between antigen-specific T cells and the antigen-presenting cells (APCs). These stable interactions would lead to T cell activation and the subsequent down-regulation of the sphingosine-1-phosphate (S1P) receptor S1P_1_ at the T cell surface, preventing antigen-specific T cells to egress the lymphoid organ (6). It was also shown that CD69 expression induced the down-regulation of S1P_1_ since cells from CD69^−^ mice failed to be retained (7, 8). However, these events only explain why antigen-specific-cells remain in contact with the APCs presenting the antigen. They do not explain how all lymphocytes dispersed throughout the body are “screened” for such binding capacity during a very short time-period after immunization. This is particularly problematic since it was shown that immediately after infection the number of APCs is very low: using the dose L50 of influenza virus in aerosols only four infectious particles were transmitted (9). In these circumstances, some circulating antigen-specific-cells may fail to contact these rare APCs, unless their transit time through the draining lymph node (DLNs) is considerably modified.

Indeed, early studies on lymphocyte trapping revealed that local recruitment of antigen-specific-cells was always preceded by profound modifications of migration affecting all lymphocytes. Elegant experiments in the sheep (3, 4) where the entry from the blood and the traffic in afferent and efferent lymphatic vessels were directly evaluated, as well as experiments in the mouse (5) showed that shortly after antigen administration, the influx of both antigen-specific and non-specific T cells into the DLN was much increased (3, 5) whereas egress was totally blocked for 1–3 days (3–5). This early reaction named “antigen non-specific trapping” or “lymph-node shut-down-phase” was considered fundamental to allow every lymphocyte enough time to move among resident cells until meeting the rare APCs first presenting the Ag. Early studies proposed that such accumulation was due to an increase in local blood flow (10), but other studies indicated that perfusion rates were not modified: the apparent increase in blood flow was only due to an increase in the size of the organ (11). Moreover, an increase in the blood flow may explain the increase in lymphocyte input, but does not explain concomitant “shut-down-phase.” Other studies reported that several inflammatory mediators could modify cell egress. Interferons (12) and TNF were reported to have these effects, but a detailed study of the effects of TNF injection showed they did not mimic those induced by antigen (13).

We here describe that CD8 T cells express a burst of pro-inflammatory cytokines immediately after cognate antigen stimulation. These inflammatory effectors follow opposite rules to classical CD8 functions, and are very potent effectors. They recruit non-resident cells, 56 cells injected into a LN recruiting up to 10^7^ cells in 24 h, including lymphocytes and multiple accessory cell types involved in inflammatory reactions. They are generated even in sterile immunizations. These results describe the characteristics of a new CD8 effector differentiation phase. They show that although local inflammation modulates T cell responses, CD8 cognate responses also initiate local inflammatory reactions.

## Materials and Methods

### Mice and immunization protocols

C57BL/6 mice expressing the CD90.1 allotype marker (Ba mice) and monoclonal (Mo) Rag2^−/−^ mice expressing TCRαβ Tgs specific for the GP33 peptide of the LCMV (P14) or for the HY antigen (HY) expressing different CD45 allotypes were obtained from our breeding colonies at the Center for the Development of Advanced Experimental Techniques, Orleans, France. CD3ε^−/−^CD45.1^+^ mice, and CD90.2^+^ Mo Rag2^−/−^ mice expressing MoTCRαβ Tg receptor specific for OVA peptides (OT1 or OT2) and MyD88-deficient mice were gifts respectively from Antonio Freitas, and from Mathew Albert and were bred at the Pasteur Institute’ animal facilities. *Listeria monocytogenesis* (LM) (expressing both the OT1 and the OT2 OVA peptides: LM-OVA) or LM-GP33 were kind gifts from L. Lefrançois – University of Connecticut Healthcare Center, Farmington, CT. For immunization with LM, sex-matched 6–8 weeks old CD90.1^+^ B6 mice were adoptively transferred with 10^6^ lymph-node cells derived from either MoP14 Tg mice or MoOT-1 Tg mice. One day later, LM were recovered during the exponential growth phase, and mice were injected i.v. with 5000 CFU LM. When specified in the text, naïve MoTg cells were labeled with 5 μM CFSE (Molecular Probes, Eugene, OR, USA) prior to injection. GP33-specific endogenous cells were obtained from wild type or MyD88-deficient mice immunized with the 5,000 CFU LM-GP33. Under both these infection conditions, bacterial loads (determined as CFU per liver or spleen) peaked at post-infection days 2–3, and the response peak was by day 8–10 after infection (not shown). For the generation of CD8 HY-specific effector cells, 6–8 weeks Rag2^−/−^ female mice were injected i.v. with a mixture of 10^6^ female and 10^5^ male bone marrow cells from CD3ε deficient mice (14). Two days later these mice were injected i.v. with 0.5 × 10^5^ CD4^+^ (Marilyn) and CD8^+^ Mo TCR-Tg cells specific for the male antigen.

### Antibodies used for flow cytometry analysis and cell sorting

The following monoclonal antibodies (MoAbs) used for flow cytometry and cell sorting were obtained from BD Pharmingen (San Diego, CA, USA): anti-CD3, anti-CD4, anti-CD8 (53-6.7), anti-CD8b (H35-172), anti-CD11b/Mac-1 (M1/70), anti-CD11c, anti-CD19, anti-CD44 (1M781), anti-CD45.2 (104-2.1), anti-CD69, anti-CD90.2/Thy1.2 (53-2-1), anti-DX5, anti-NK1.1 (PK136), anti-Ly6G/Gr1 (RB6-8C5), anti-Ly6c, anti-PDCA-1. All the above-mentioned mAbs were directly coupled to FITC, PE, PerCP, PECy7, allophycocyanin or Pacific Blue, or conjugated with biotin. Biotinylated mAbs were revealed with streptavidin-allophycocyanin (BD Pharmingen, San Diego, USA), or streptavidin-Pacific Orange (Molecular Probes, Eugene, USA). Innate cell populations present in brachial lymph node (BRLN) after the injection of naïve or effector cells were defined as following: NKs: DX5^+^ NK1.1^+^; cDCs: CD11c^high^PDCA-1^−^; pDC: CD11c^low^PDCA-1^+^; monocytes: CD11b^high^ LyC6^high^; granulocytes (PMNs): CD11b^high^Ly6C^low^. For the *ex vivo* detection of cytokines and chemokines, mice were injected with 0.25 mg of Brefeldin A (Sigma-Aldrich, St. Louis, USA) and intracellular staining performed 6 h later (15), with the following Abs: rat anti-mouse CCL3 (clone IC450A, R&D Systems, Minneapolis, MN, USA); rat anti-mouse TNF-α (clone 557644, BD Pharmingen, San Diego, CA, USA), rat anti-mouse CCL4 (clone MAB451, R&D systems). Antibodies for phosphorylated signal transduction molecules and the respective isotype controls were purchased from Cell Signaling Technology (Danvers, MA, USA): Akt (Ser473, clone D9E)-PE, NF-kB p65 (Ser536, clone 93H1)-Alexa Fluor 488, p44/42 MAPK (Thr202/Tyr204, clone E10)-Alexa Fluor 488, p38 MAPK (Thr180/Tyr182, clone 28B10)-Alexa Fluor 647 and SAPK/JNK (Thr183/Tyr185, clone G9)–PE. Cells were analyzed on a FACSCanto system and sorted on a FACS Aria system (Becton Dickinson, Franklin Lakes, NJ, USA).

### Quantification of antigen-specific endogenous cells

All the individual steps of this method are required to achieve optimal recovery and quantification of naïve cells. Organs were totally cleaned of fat and other adjoining tissues and distributed in 24-well plates in RPMI medium supplemented with 2% fetal calf serum and HEPES buffer. Cell suspensions were obtained by mechanical disruption with forceps followed by digestion with 0.5 mg/ml collagenase type IV (Worthington Biochemical Corporation, Lakewood, NJ, USA) and 5 μg/ml deoxyribonuclease I (Sigma-Aldrich, St. Louis, MN, USA) for 30 min at 37°C in 5% CO2 with agitation. We found that this digestion step was critical, since cell yields were much higher and the resulting cell suspensions cleaner when compared with those obtained by mechanical disruption alone.

For counting GP33-specific naïve cells, a known number of LN Mo P14 Tg cells expressing different allotypes were added directly to these suspensions prior to any further manipulation. The cells were then washed and depleted of non-CD8 T cells with a cocktail of MoAbs (TER119, CD19, Mac-1, GR1, CD4, B220) and Dynabeads (Dynal AS, Oslo, Norway). All these Abs were previously titrated to determine the binding efficiency and the absence of non-specific binding/depletion. We found that this enrichment step was required to optimize the labeling and discrimination of endogenous antigen-specific-cells. Cells were labeled with PE- and APC-labeled multimers of MHC class I loaded with GP33 peptide (Dextramers^®^, Immudex, Copenhagen, Denmark) previously titrated on P14 Tg cells, and antigen-specific-cells recovered by pull-down (16). In contrast to MHC tetramers, these multimers associate a higher number of fluorochrome and peptide-loaded MHC class I molecules, enabling a better discrimination between antigen-specific and non-specific endogenous cells. For counting antigen-specific-cells, the labeled populations were diluted in 0.5–1 ml of FACS flow buffer and acquired using the low-speed mode in a FACS-Canto. The use of low-speed mode was also found important, since it reduced both the cell loss during acquisition and the background non-specific labeling.

### Cytokine expression. Correlations between cell differentiation and division “*in vivo*”

Assessment of the correlations between the expression of several pro-inflammatory mediators and molecules involved in cytotoxicity with division *in vivo* was hindered by (i) the very low number of cells present at each division at early time points of the response and (ii) the small number of parameters than can be reliably used with CFSE. In order to overcome these difficulties and study the expression of several effector molecules simultaneously, we used a single-cell multiplex RT-PCR technique previously developed in our laboratory (17). This technique allows the simultaneous detection of the expression of *Tgfb1, Tnf*, and cytotoxic genes in each individual cell and we created compatible primers for the simultaneously detection of chemokines (Figures S1 and S2 in Supplementary Material). Briefly, individual cells were sorted and lysed and the mRNA reverse-transcribed by using specific 3′ primers for all genes. The 5′ primers are then added, and a first 15-cycle amplification step is initiated. The PCR products are then split into different wells and a second, nested PCR is performed for each gene separately. Given that the primers amplifying all the mRNAs are present in the first PCR, the arrays have two major requirements. Firstly, all PCRs should have the same efficiency, to prevent preferential amplification. Secondly, neither the primers nor the amplicons should compete during the first PCR reaction. For new primers, validation of these two requirements are shown in Figures S1 and S2 in Supplementary Material. Validation of the other primers has been described previously (17).

The following primers were selected:

*Xcl1*: forward 5′-GAC-TTC-TCC-TCC-TGA-CTT-TC-3′, nested forward 5′-GGA-CTG-AAG-TCC-TAG-AAG-AG-3′, and reverse 5′-TGC-CAT-CCA-CAG-TCT-TGA-TC-3′
*Ccl3*: forward 5′-AAG-GAT-ACA-AGC-AGC-AGC-GA-3′, nested forward 5′-CCA-GTC-CCT-TTT-CTG-TTC-TG-3′, and reverse 5′-GAT-CTG-CCG-GTT-TCT-CTT-AG-3′
*Ccl4*: forward 5′-CCA-GCT-CTG-TGC-AAA-CCT-AA-3′, nested forward 5′-GAG-CAA-CAC-CAT-GAA-GCT-CT-3′, and reverse 5′-GCT-CAG-TTC-AAC-TCC-AAG-TC-3′
*28S*: forward 5′-TAC-CGG-ACC-CTG-AAC-AGA-AT-3′, and reverse 5′-GAT-GAT-CCT-CCG-GCA-TGT-TT-3′ (28S amplification was used to test the plating efficiency).

We used two independent approaches to show that this method can detect as few as two mRNA molecules per cell (17). *NB*: the expression of all these effector molecules requires cognate antigen stimulation. Naïve cells do not express these mRNAs. The infection milieu does not induce the expression of these mediators: when mice are infected with LM that does not express OVA, OT-1 cells do not express these mediators.

Detection of cytokine proteins *ex vivo* was performed after injection of Brefeldin A, as described (15).

### The inflammatory capacity of CD8 T cells *in vivo*

Sorted naïve or effector CD90.2 Tg T cells obtained from the spleen 2.5 days after immunization. In some experiments they were mixed at 4°C with 50% “High Concentration” Growth factor-reduced Matrigel (BD Biosciences, San Jose, USA), and injected subcutaneously in the ear of CD90.1 recipient mice. In other experiments, the BRLN was accessed by a small vertical skin incision parallel to medial border of the scapula in anesthetized mice. The same cells were injected directly in the BRLN in a 10 μl volume using an insulin syringe, and the skin incision was closed with one wound clip. We aimed to inject 60 or 600 cells/mouse but variations in cell numbers were to be expected after such high dilutions and injection. To determine the actual number of cells present in the BRLN after injection, we counted them in the final cell suspension and in the BRLN of control mice shortly after injection.

To determine the best time point for studying the effect of these effectors, we measured the weight of LNs at different times after injection of effector T cells. For evaluation of S1P, LNs were studied 16 h after the injection of effectors, since their weight had not yet increased at that time point. For the detection of cell accumulation, LNs were studied between 24 and 30 h post-injection, when they had already increased in size. For the detection of S1P, we used the S1P bioassay described previously (18) with WEHI231 cells expressing Flag-S1P1 (a kind gift of J. Cyster). This method was slightly modified to increase sensitivity. To concentrate the S1P amounts recovered from the LN, we extracted S1P in a 50 μl volume, and reduced the reaction volumes to 40 μl and the number of WEHI231 cells to 2 × 10^4^/well. To reduce background labeling (variations of Flag-S1P1 expression in WEHI231 cells incubated with medium alone) these cells were previously sorted to obtain homogeneous populations expressing the same level of Flag-S1P1.

## Results

### CD8^+^ inflammatory effector T cells do not follow the rules thought to govern CD8^+^ differentiation into effector cells

**Figure 1:**
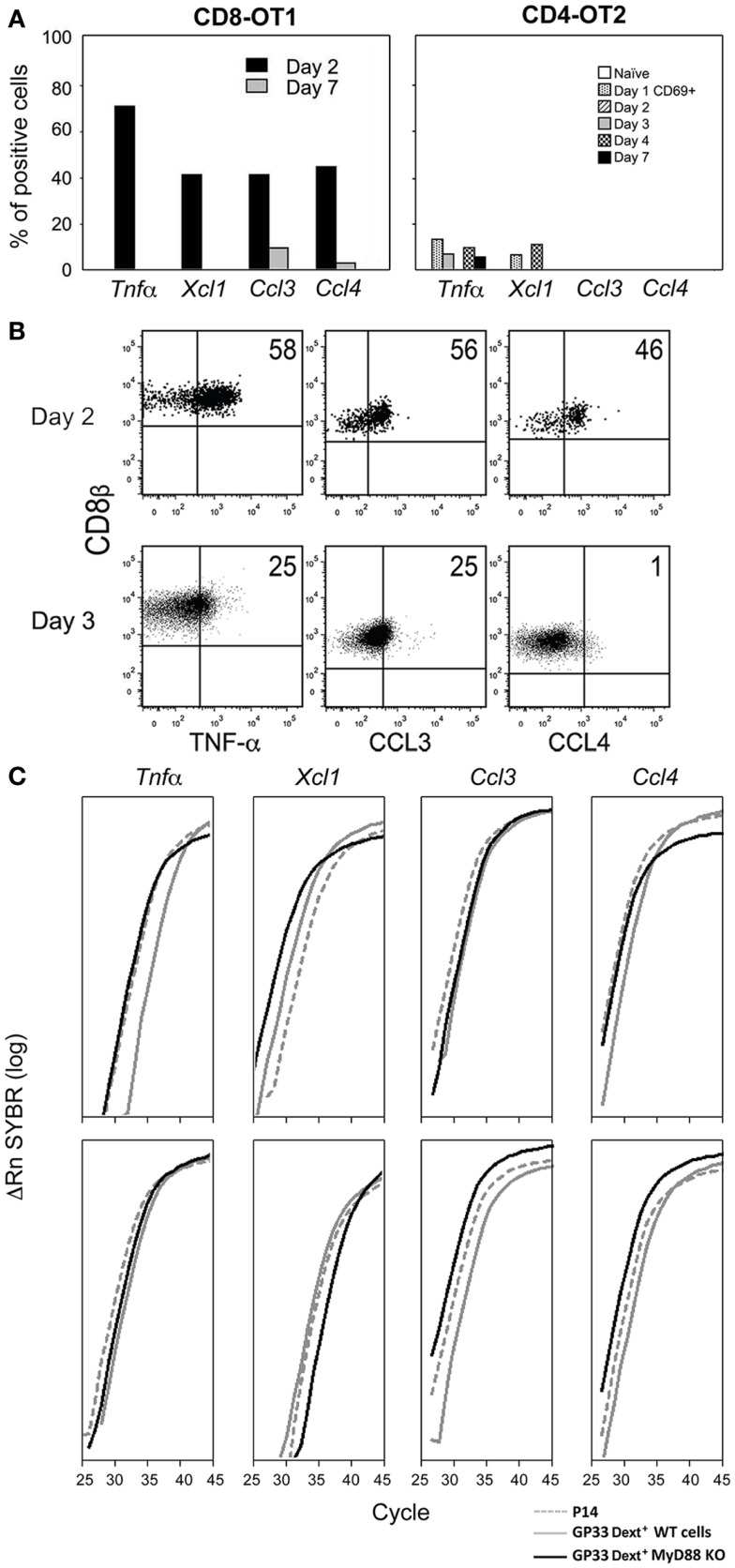
**Expression of inflammatory mediators by CD8 T cells**. **(A,B)** Mo CD90.2 TCR-Tg cells were transferred to CD90.1 B6 mice, and immunized with LM-OVA expressing the OT1 and OT2 epitopes. At different time points after infection we determined: **(A)** The proportion of OT1 and OT2 Tg cells expressing these mRNAs evaluated in 72 individual cells by single-cell RT-PCRs. **(B)** The *ex vivo* intracellular expression of pro-inflammatory proteins in Brefeldin A injected mice. Barriers to identify positive cells were based on the labeling of the same cell suspension with isotype control antibodies. The lack of suitable Abs prevented us from evaluating XCL1 expression. **(C)** Mo CD90.2 P14 TCR-Tg cells were transferred to CD90.1 B6 mice. 24 h later, these transferred mice, as well as My88^+^ and MyD88^−^ mice were immunized simultaneously with LM-GP33. CD8^+^ CD69^+^ GP33-specific-cells were sorted from these three mice types at days 2 and 3 after priming. For that purpose, spleen cell suspensions were depleted of non-CD8 T cells. The P14 Tg cells were identified by their co-expression of CD90.2 and CD8β. Endogenous cells from MyD88^+^ and MyD88 B6^−^ mice were identified by triple co-expression of CD8β, and APC, and PE labeled GP33-Dext. Results show the levels of these mRNAs in CD69^+^ GP33-specific-cells 2 (upper graphs) and 3 days (lower graphs) after immunization, evaluated by qRT-PCR.

Since in the first 1–4 days after antigen administration cell input to the DLN is much increased, we studied if CD8 T cells would express mediators justifying this increase. When Mo TCR-Tg cells were stimulated with LM expressing the OT1 and the OT2 epitopes, CD8 OT1, but not the CD4 OT2 cells abundantly expressed a panoply of chemokines – *Xcl1, Ccl3*, and *Ccl4* by day 2 after immunization, while this expression was lost before the immune response peak (Figure 1A). OT1 cells also expressed *Tnf* (Figure 1A), reported to increase HEV permeability and reduce LN egress (19). *Ex vivo* detection of protein in Brefeldin A injected mice confirmed that secretion of pro-inflammatory mediators was restricted to the early response (Figure 1B). Production of these mediators was dependent on the recognition of the Ag by antigen-specific-cells. When these mice were injected with LM not expressing OVA, OT-1 cells did not express any effector function. Mo CD8 anti-HY TCR-Tg cells but not Mo CD4 anti-HY Marilyn TCR-Tg immunized with male cells also produced a similar cytokine/chemokine burst, although *Ccl3* and *Ccl4* levels were lower than found in OT1 CD8 cells (not shown).

Since the above results were obtained with TCR-Tg cells we wished to evaluate if endogenous cells shared the same properties. Moreover, since immunization with male cells does not involve obvious innate signals we wished to study the impact of such signaling in inflammatory effector functions. We compared by qRT-PCR the expression of the mRNAs coding for these mediators by different types of CD69^+^ GP33-specific CD8 T cells, immunized with LM-GP33. We found equivalent expression in TCR-Tg cells from P14 mice and in endogenous cells from MyD88^+^ or MyD88^−^ B6 mice (Figure 1C). These results show that endogenous and TCR-Tg cells share the same properties. Moreover, such properties are not affected by the abrogation of the MyD88 pathway.

**Figure 2:**
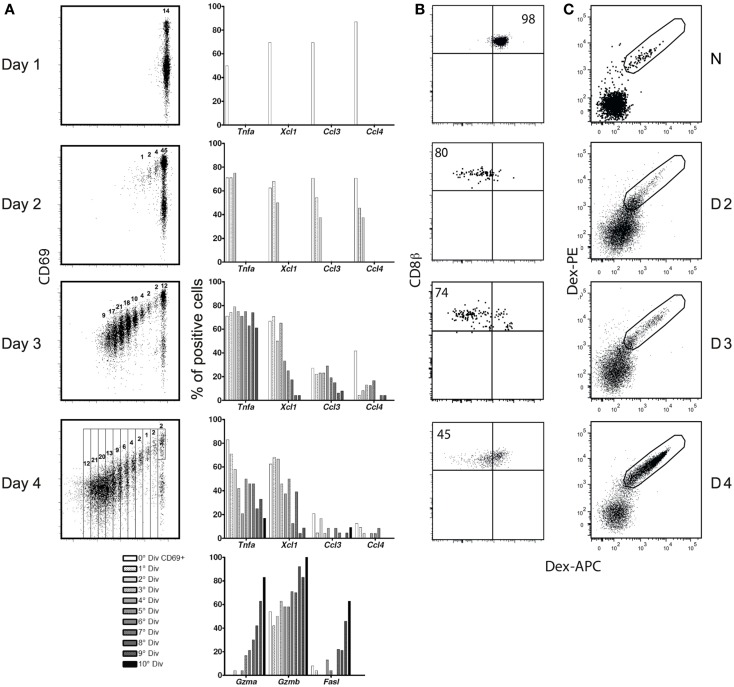
**(A)** Correlation between CD8 division and differentiation. **(A)** CFSE-labeled Mo CD90.2 OT-1 Tg cells were transferred to CD90.1 B6 mice and studied at different time points after infection with LM-OVA. Left: CFSE dilution and the gates used to sort individual cells at each division. Right: expression frequency, as evaluated in RT-PCRs in 96 individual cells. **(B,C)** TCR expression after immunization with LM-GP33. **(B)** TCR-Tg cells. 5,000 Mo CD90.2^+^ P14 TCR-Tg cells were injected into CD90.1^+^ B6 mice prior to infection. Results show APC-Dext-GP33 binding in CD90.2^+^ CD8^+^ Tg T cells in naïve (upper graph) and infected mice, at different days (D) after immunization. **(C)** Endogenous cells. CD90.1 B6 mice were immunized with LM-GP33. Results show Dext-GP33 PE and APC double-labeling in gated CD8 spleen cells in naïve mice (upper graph) and primed mice at different days (D) after infection.

The kinetics of expression of these mediators was surprising, since it is generally believed that all CD8 effector functions increase with cell division, and decline when antigen is eliminated (20, 21). Correlation of cell division with differentiation (Figure 2A) confirmed that the expression of cytolyic effector molecules does follow these rules: they increase with division (lower panel), and we previous confirmed that they decline only after antigen elimination (2). In contrast, inflammatory mRNAs were expressed in CD69^+^ cells before any division, declining with division. While effector functions are believed to decline only when antigen is eliminated, inflammatory effectors also contradicted this rule, as they were lost by day 4 after immunization (Figure 2A), when we previously showed that antigen concentrations were still (2).

**Figure 3:**
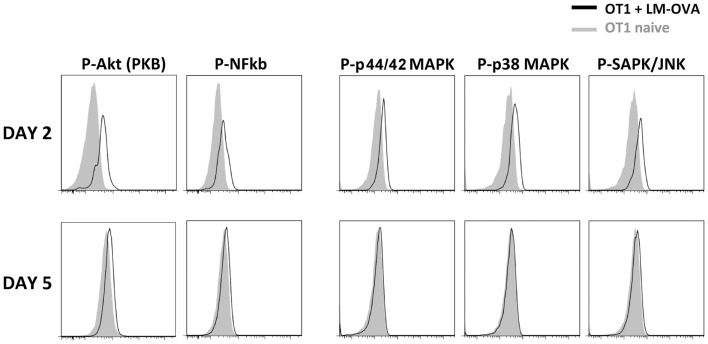
**TCR signaling transduction at different time points after immunization**. Mo CD90.2^+^CD45.1^+^ OT-1 TCR-Tg cells were transferred into CD90.1^+^ CD45.2^+^ B6 hosts and infected with LM-OVA. The figure shows the expression of phosphorylated signal transduction molecules in CD90.2^+^ CD45.1^+^ CD8^+^ Tg cells at different time points after priming. The labeling with isotype controls is shown in gray.

We next wished to determine the cause for this early decline. Given that the expression of these mediators requires MAPK activation, we looked at whether such activation could be interrupted during the early phases of the CD8 response. Indeed, both OT-1 TCR-Tg cells and endogenous cells down-regulated their TCR (Figures 2B,C) and CD3ε (not shown) losing the ability to bind peptide-loaded dextramers (Dext: MHC multimers with a higher number peptide – MHC complexes than tetramers). The MAPK activation declined progressively and was fully abrogated before the immune response peak (Figure 3), restricting CD8s’ inflammatory profile to the first days after priming.

Overall, these results demonstrate that the classic description of the sequential steps of CD8 responses (activation, expansion, differentiation into effector functions, antigen elimination, effector function loss) must be revised. CD8 T cells actually undergo two independent differentiation phases, governed by opposite rules. Immediately after activation they differentiate into inflammatory effectors, but then lose their pro-inflammatory properties as they divide and gradually differentiate into CTLs.

### The physiologic role of CD8^+^ inflammatory effector T cells: I. Quantification of their number

Since the generation of inflammatory effectors is restricted to the first three-four divisions, their number should never exceed that of naïve cells by more than a factor of eight. It is difficult to envisage that such low effector number has any major role in the immune response, since the number of naïve cells for a single GP33 epitope was described to average 200 cells/mouse (16). However, this number is known to be an underestimate (16). Indeed, from the 50 LN reported to be present in the mouse, only nine were included in this calculation. The identification of the naïve cells after tetramer pull-down was not controlled for the efficiency of tetramer binding nor for losses during purification.

We revised these estimates by treating individual organs with collagenase/DNase (increasing substantially total cell recovery), and adding a known number of antigen-specific “reference populations” (RP) to these cell suspensions. These RP bind the same tetramers as endogenous cells and therefore can be used as controls for the efficiency of tetramer binding. Since they undergo the same purification steps, their recovery rate can be used to control non-specific cell losses during purification. We also used the more efficient Dext instead of tetramers for the purification of antigen-specific-cells. Using these combined approaches, we evaluated the total number of naïve cells for the LCMV response. This evaluation can only be determined for this response, since this calculation requires the identification of all antigen epitopes and their relative representation in the response. These data are only available for the LCMV response, where it is known that the GP33 specificity corresponds to 10% of the LCMV specific-cells (22).

**Figure 4:**
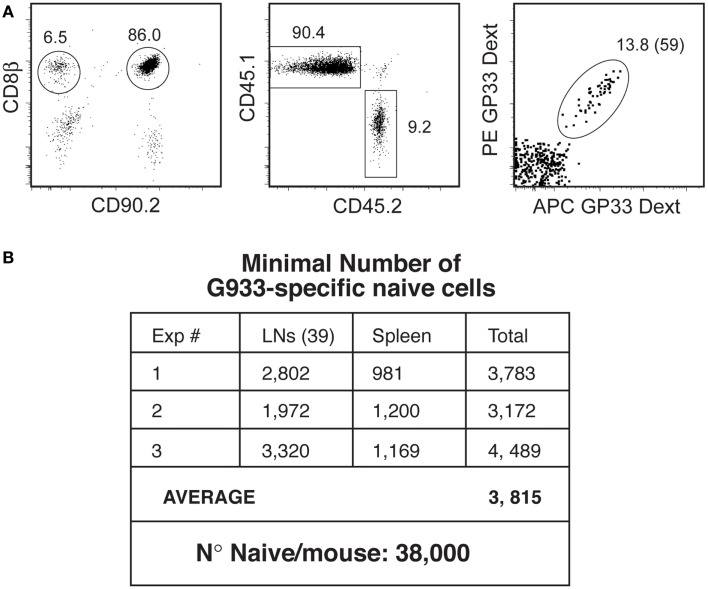
**Quantification of the naïve T cell pool**. **(A)** Quantification of antigen-specific naïve cells in the BRLN. Reference populations (RPs: 10^4^ CD90.2 CD45.1 cells and 10^3^ CD90.2 CD45.2 P14 cells) were added to collagenase/DNase digests of the BRLN from CD90.1 B6 mice. Cell suspensions were depleted of non-CD8 T cells, and antigen-specific-cells purified by Dext pull-down. Left: the gates used to identify CD90.2^+^ RP and CD90.1 endogenous cells. Middle: CD45 allotypes distribution in RPs. Right: GP33-Dext labeling in CD90.1 endogenous CD8 T cells. **(B)** Estimation of the size of the naïve GP33-specific T cell pool. Results show the number of Dext-GP33-specific-cells recovered/mouse in three independent experiments.

When two RP of monoclonal P14 TCR-Tg LN T cells (10^4^ CD90.2 CD45.1 and 10^3^ CD90.2 CD45.2 P14 cells) were added to BRLN of CD90.1 CD45.2 mice, endogenous CD90.1 CD8 T cells could be easily differentiated from CD90.2 RP populations (Figure 4A: left). Both RP (CD45.1 and CD45.2) were fully labeled with GP33-Dext, demonstrating that Dext binding of the suspension was efficient (not shown). Moreover, CD45.2 and CD45.1 RP populations maintained the same relative representation after purification (10:1) demonstrating that less abundant cells were not preferentially lost (Figure 4A, middle). Within CD90.1^+^ endogenous CD8 T cells, Dext^+^ cells were clearly visualized (Figure 4A, right), the number of GP33-specific-cells in the BRLN averaging 60. Similar results were obtained for the number of epitope HY-specific or SIINFEKL – OVA specific naïve CD8^+^ cells.

To calculate the number of naïve cells/mouse identical studies were performed in the spleen and in 39 other LN. In the three independent experiments we performed we found about 3,800 naïve GP33-specific-cells (Figure 4B). Since the GP33 specificity corresponds to 10% of the LCMV response (22), total number of naïve cells responding to LCMV should be about 3.8 × 10^4^, and the total number of inflammatory effectors could reach 3.04 × 10^5^. It must be noted that this number is still an underestimate, since we only managed to recover 39 LN of the 50 LN described, and naïve cells present in the blood and bone marrow were not counted. However, it is likely that the number of naïve cells would not be much higher since the LN we did not harvest were described as relatively small, and the BM harbors mostly antigen-experienced cells.

### The physiologic role of CD8^+^ inflammatory effector T cells: II. Chemokines can attract cells at distance

**Figure 5:**
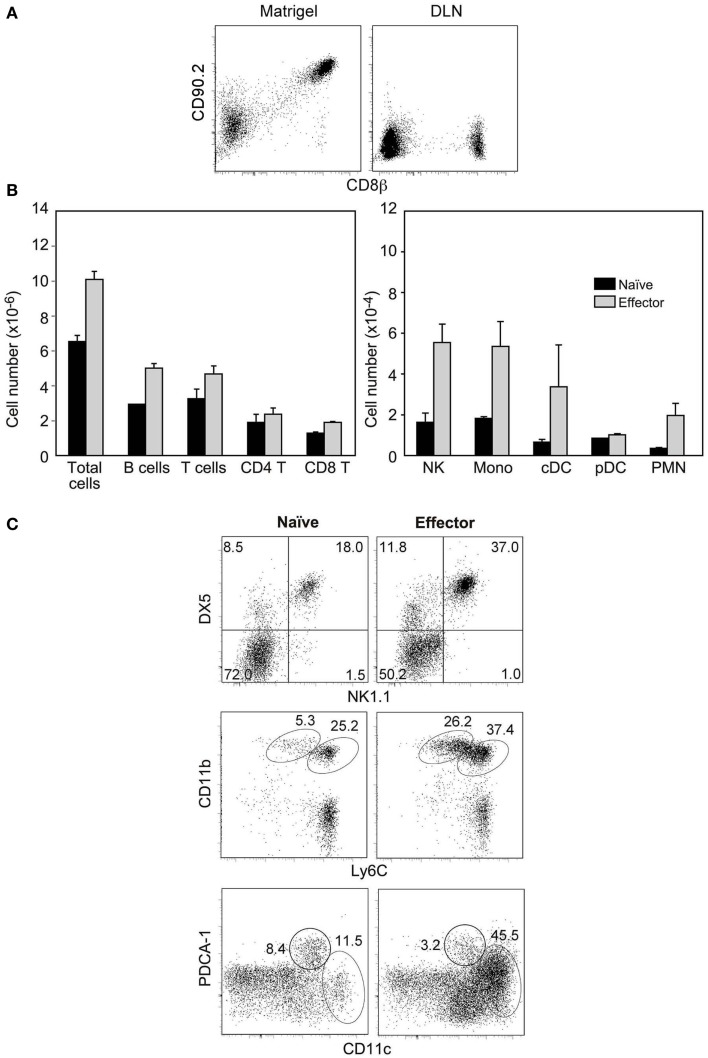
**Inflammatory effector CD8 T cells recruit non-resident cells**. Sorted Mo CD90.2^+^ OT-1 naïve cells or inflammatory effectors (recovered 2.5 days after LM-OVA infection) were injected with matrigel subcutaneously in the ear of CD90.1^+^ mice. **(A)** Recovery of injected cells from the matrigel plug (left) and the auricular LN (ALN) (right) 24 h after injection. **(B,C)** Cell populations recovered in the ALN of injected mice, 24 h after injection. **(B)** Results show cell numbers and are mean±SEM of three independent experiments. **(C)** Results show the gates used to identify different cell types and are from one representative experiment out of the three we performed.

A role of the chemokine burst produced by CD8 T cells in the increase in cell input during antigen-non-specific trapping would imply that these chemokines should create a gradient able to recruit cells located out-side the DNL. However, it is yet unclear if chemokines only modify the migration of resident cells (23). To test if CD8 inflammatory effectors attract cells at distance, they should be isolated from the *in vivo* inflammatory milieu, transferred to a normal mouse and retained in a particular site where non-resident cells would not migrate, and their effect studied at distance. We found that this was possible when sorted effectors were immobilized subcutaneously in the ear with the collagen-laminin matrix Matrigel. Matrigel is liquid at 4°C but solidifies after injection, and is frequently used to promote viability of tumor cell lines injected subcutaneously. When injected with Matrigel, effectors remained viable and at the injection site and could be recovered and from the Matrigel plug, but were absent in the auricular LN (ALN) (Figure 5A) and other organs (not shown) 24 h later. When 5 × 10^4^ naïve OT-1 were immobilized in the ear, naïve cells did not modified the number or cellular composition of the ALN as compared to non-injected mice (not shown). In contrast, when similar numbers of OT1 inflammatory effectors were retained, the ALN increased in size, accumulating both T and B lymphocytes (Figure 5B). We also observed a preferential accumulation of NK cells, CD11b^+^Ly6C^hi^ inflammatory monocytes, cDCs, and CD11b^+^Ly6C^low^ PMNs (Figures 5B,C) reflecting the production of MIP-1 and XCL1 chemokines by these CD8 T cells. Similar effects were observed when the effector number was reduced 20-fold. These results show that CD8 inflammatory effectors are able to recruit non-resident cells.

**Figure 6:**
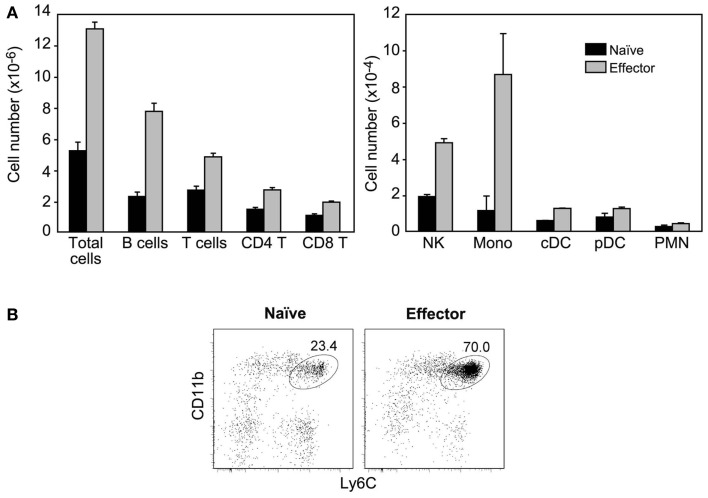
**CD8^+^ inflammatory effector T cells generated in sterile immunizations also recruit non-resident cells**. Sorted Mo CD90.2^+^ HY-specific CD8 naïve cells or inflammatory effectors (recovered at 2.5 days after immunization with male cells) were injected with matrigel subcutaneously in the ear of CD90.1^+^ mice. **(A)** Results show the number of different cell types recovered in the ALN of injected mice, 24 h after injection and are the mean±SEM of three independent experiments. Cell populations were identified as described in Figure 5. **(B)** Major accumulation of inflammatory monocytes in the ALN of mice injected with inflammatory effectors. Results are from one representative experiment, *out of the three performed*.

When immobilized in the ear, HY naïve cells had no effect, while HY-specific inflammatory effectors induced a major accumulation of XCR1 expressing cells (24, 25): T and B lymphocytes, NK cells, and CD11b^+^Ly6C^hi^ inflammatory monocytes. Indeed, upon injection of HY effectors the ALN could triple in size, i.e., LN hypertrophy was more marked than that found after injection of OT-1 effectors. Accordingly to their lower expression of MIP-1 chemokines, DCs and PMNs that are recruited by MIP-1 chemokines (26, 27), were less affected (Figure 6). Thus, CD8 inflammatory effectors generated after a sterile immunization are also able to recruit non-resident cells at distance.

### The physiologic role of CD8^+^ inflammatory effector T cells: III-Local modifications after intra-nodal injection of physiologic numbers

**Figure 7:**
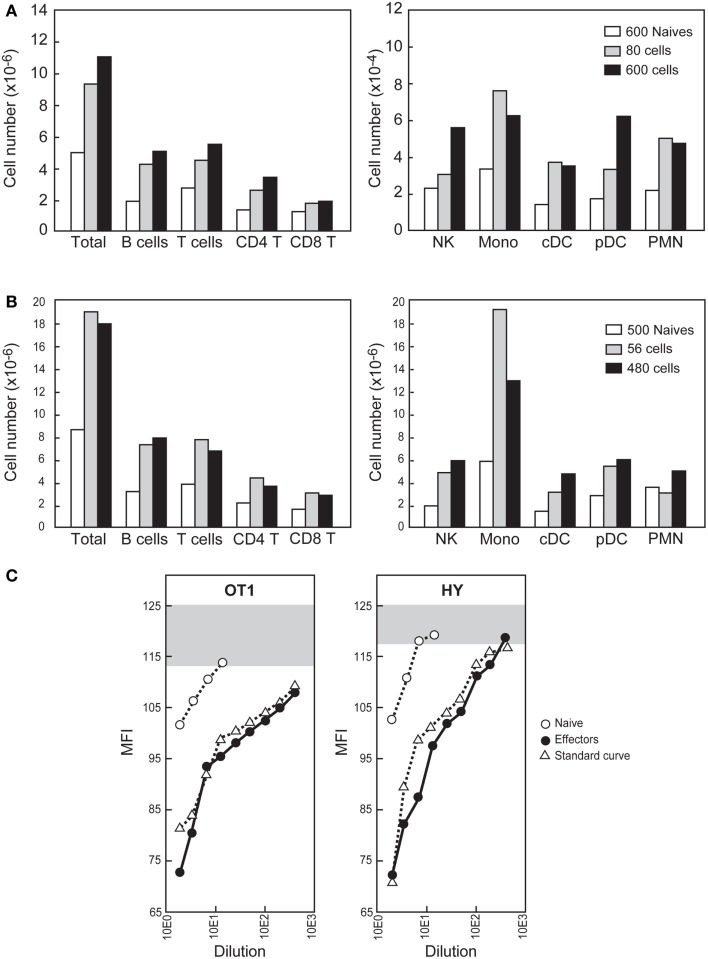
**Effects of intra-nodal injection of physiologic numbers of CD8^+^ inflammatory effector T cells**. **(A,B)** The indicated number of sorted CD90.2^+^ naïve or inflammatory effectors (recovered 2.5 days after priming) were injected into the BRLN of CD90.1^+^ B6 mice. The BRLNs were removed 24 h later and the different cell types identified as shown in Figure 5. Results compare cell recovery in mice injected with naïve CD8+ T cells or inflammatory effector CD8^+^ T cells **(A)** Mice were injected with OT-1 Tg cells. **(B)** Mice injected with HY Tg cells. Similar results were obtained after injection of P14 Tg cells. **(C)** The BRLNs were injected with 500 naïve cells, 80 OT-1 effectors or 56 HY effectors. Results show of S1P amounts in LN lysates 16 h later, compared with known amounts of a synthetic S1P. Gray bars represent the Flag-S1P1 expression of WEHI231 incubated with medium alone. Results are representative of the seven experiments we performed.

Since at the beginning of a natural infection the inflammatory effectors should only be the rare resident cells already present in a lymphoid organ, we aimed to study the role of such effector numbers after intra-nodal injection. We first selected the target LN used for injection, which should be relative large (facilitating calculation of the number of naïve resident cells) and giving easy access to injection. We studied the positions and sizes of several superficial LNs and the best procedure to access them and found the BRLN suitable in both respects. After collagen and DNase digestion it harbors 5–8 × 10^6^ cells. It can be reproducibly accessed through a very small skin incision near the scapula. Since in this node the number of naïve cells specific for the GP33 epitope averages 60 cells (Figure 4A), the number of naïve LCMV specific-cells should be 600, since the GP33 specificity corresponds to 10% of the LCMV response (22). Therefore the total number of resident effectors should be lower than 4,800. Remarkably, even a much lower number of OT-1 (Figure 7A) or anti-HY inflammatory effectors (Figure 7B) injected directly in the BRLN induced a major local recruitment. Again, the type of recruited cells reflected the chemokine profile expressed by each effector type.

We were surprised that as few as 56 effectors could attract up to 10^7^ cells to a LN in 24 h (Figure 7B), and wondered whether other mediators could be involved. Given that (i) LN egress is totally blocked during early trapping (3, 4) and (ii) S1P gradients control this egress (18), we hypothesized that inflammatory effectors could increase local S1P concentrations and thus contribute to the local retention. Indeed, since inflammatory mediators induce the release of S1P by certain resident cell lines *in vitro* (28, 29), it was hypothesized that local inflammation could induce S1P release by tissue-resident cells and thus contribute to cell accumulation at the inflammatory site (30). This attractive hypothesis could not be confirmed experimentally since previous methods did not allow S1P measurements in small tissue fragments. We modified these methods to increase sensitivity and found that intra-nodal injection of inflammatory effectors induced the up-regulation of S1P in tissue extracts from that BRLN 16 h later (Figure 7C). By comparison with a known concentration of standard, synthetic S1P, the amounts of S1P recovered from BRLNs injected with effectors ranged from 1422 ng/g to about 2800 ng/g, i.e., 2.4- to 5-fold higher than the value reported after direct extraction of S1P from a large LN pool (18). This value is likely an underestimate, since the S1P levels in BRLNs injected with 56 effectors were 10-fold higher than in BRLNs injected with a 600 naïve cells, studied simultaneously (Figure 7C). Since a twofold increase in the S1P concentrations is enough to induce major changes in lymphocyte egress (6) the S1P amounts detected here justify the blockage of LN egress.

## Discussion

Our present results characterize an important function of CD8 T cells, which modifies the actual paradigms on: (i) when CD8 cells acquire and lose effector functions; (ii) the roles that CD8 cells have in immune responses; and (iii) how inflammation and cognate immune responses interact.

It is generally believed that after antigen stimulation CD8 T cells effector functions increase with division and decline once the antigen is eliminated. Our results allow comparing the differentiation into killer or inflammatory functions by the same CD8 population throughout divisions/throughout time. While cytotoxicity follows the “classic” rules of CD8 differentiation, opposite rules govern CD8 inflammatory functions. These are induced before division, decrease while cells divide and decline at the time point of the response when we previously showed antigen concentrations to be quite high (2). The shortness of this inflammatory burst is likely due to extensive TCR down-regulation, which abrogates MAPK activation preventing the expression of these mediators.

The loss of TCR surface expression after *in vitro* T cell activation is well documented, but it was shown to reverse by 24 h (31). Down-regulation of the TCR was also reported after *in vivo* immunization (32, 33). However, we were now able to quantify this phenomenon. By visualizing Mo TCR-Tg cells recognizable by a different allotype, present in frequencies equivalent to those of endogenous antigen-specific-cells, we found that 80% lost their TCR expression by day two after priming. Moreover, this down-regulation persisted for several days: by day 4 after immunization 50% of the responding cells yet fail to express the TCR. Comparison of Dext labeling in naïve and primed endogenous cells also shows considerable down-regulation during these time points. These results identify a major limitation to the study of endogenous CD8 responses. They show that the majority of endogenous antigen-specific-cells cannot be identified during early responses.

While it is generally believed that the major CD8 effector functions are γ-IFN production and cytotoxicity, we now describe in detail a new CD8 effector phase: the secretion of a major burst of pro-inflammatory mediators shortly after activation which directly or indirectly promote the recruitment of lymphocytes and accessory cells types to the location where antigen is present. These cells are likely involved in the “antigen non-specific/shut-down-phase” of lymphocyte trapping-a process proposed to have a fundamental role in allowing rare APCs at a restricted location to screen the total lymphocyte pool in 24–48 h, in order that all antigen-specific-cells dispersed throughout the body may be selected for APC binding. Elegant experiments performed 4–5 decades ago identified a major increase in cell input shortly after antigen stimulation, and a total block in cell egress, which retained both antigen-specific and non-specific T cells at the location where antigen is first presented (3–5). It was proposed that these modifications were induced by an increase in blood flow (10), but our previous measures of blood flow during trapping indicated that the reported increases were due to an increase in organ size. Indeed, when the blood flow was related to the organ weight, perfusion rates were not modified (11). In contrast, our results implicate the chemokine burst produced by inflammatory effectors in this recruitment. This burst is able to mobilize non-resident cells. Importantly, the cell types recruited either at distance or after the intra-nodal injection of inflammatory effectors correlate with the chemokine profile of effector cells: HY effectors which express mainly XCL1 recruiting predominantly inflammatory monocytes and lymphocytes, while OT-1 effectors secreting CCL2 and CCL3 also promoting DC and granulocyte recruitment. Inflammatory effectors also secrete TNF, known to participate in DC and T cell recruitment besides having other functions in DC stimulation, T cell co-stimulation and survival [review in Ref. (19)].

When we first detected these early effector functions we were unconvinced that they would have any relevant role in immune responses, since the number of these effectors should be very low. Surprising, they are very potent effectors since 56 present in a LN harboring 5–8 × 10^6^ cells are able to recruit up to 10^7^ cells in 24 h. It must be noted that the different cell types recruited to the DLN may co-participate in this recruitment, since they are also able to secrete these mediators. In particular, injection of antigen-loaded DCs was shown to increase LN cellularity, which was potentiated by co-injection of TNF (34). However, it was not established if this effect was direct, or mediated by the recruited antigen-specific T cells. In spite of the possible contribution of other cell types, the time course of cell recruitment and LN “shut-down” phase overlaps that of the CD8 inflammatory burst, indicating that the latter has a fundamental role in the overall regulation of these phenomena.

Besides secreting inflammatory mediators, the intra-nodal injection of very low effector numbers also induced a 10-fold increase in S1P recovery from the injected LN, as compared to LN injected with eightfold more naïve cells. S1P up-regulation precedes LN hyperplasia, since it is already detected at 16 h after effector injection, and thus may also contribute to the local cell accumulation by immobilizing recruited cells. The kinetics of S1P up-regulation also mimics the CD8 inflammatory burst, since it is stable at 24 h and declines by days 2–3 after effector injection (data not shown). It must be noted that we studied both intracellular and secreted S1P, since it is not possible to discriminate soluble S1P from tissue samples (J. Pereira, personal communication). However, it is likely that part of this S1P is secreted, being involved in the LN “shut-down” phase of lymphocyte trapping, since (i) a twofold increase in secreted S1P was found sufficient to modify lymphocyte migration (6); and (ii) the injection of FTY720 (which is rapidly phosphorylated *in vivo* generating an SIP analog) mimics the DLN shut-down-phase (23). Part of the S1P may also be intracellular, where it was shown to have important functions, by co-participating in TNF signaling, activating NF-Kb, and contributing to the anti-apoptotic role of TNF (35).

Concerning the production of S1P, we confirmed that T cells do not secrete it (23). We failed to detect this molecule in supernatants of activated T cells *in vitro*. Quantitative evaluation of *Sphk1* and *Sphk2* showed equivalent *ex vivo* expression levels in naïve and inflammatory effectors. The S1P recovery was similar in LN injected with 56 or 1,000 effectors (not shown). Therefore, as described in all the systems studied so far, tissue-resident cells likely produced this mediator (23). In contrast, our data does not support the general believe that the TNF/SIP pathway is always responsible for S1P production and cell recruitment during inflammation. This notion issued from the demonstration that TNF induces the up-regulation of *Sphk1* expression in certain cell lines *in vitro*. We could not confirm the role of this pathway in lymphocyte trapping, since blockage of TNF activity by Abs did not modify S1P recovery in LN injected with inflammatory effectors (not shown). A detailed comparison between the effects of TNF versus antigen injection on lymphocyte migration also reported that TNF does not recapitulate the effects of antigen (13). The complexity of sphingolipids role and the regulatory mechanisms governing their metabolism is yet being revealed, but these studies are yet seriously handicapped by the low sensitivity of the methods for sphingolipids detection.

We were surprised that CD4 T cells reportedly producing MIP-1 chemokines to recruit CD8 T cells to provide help (36) had no equivalent functions. It is possible that the relative roles of CD4 and CD8 T cells in recruitment depend of the immunization context. MIP-1^+^ CD4 T cells were detected after immunization with peptide and complete Freund’s adjuvant, whereas we here studied CD8-dependent responses. Moreover, it should be noted that MIP-1 chemokines only attract previously activated T cells (37) and thus are unlikely to be involved in the recruitment of naïve cells during trapping.

Our results also modify perspectives on the putative relationships between inflammation and immune responses. Inflammation is generally regarded as starting with an innate immune response that is mediated by tissue-resident cells (38, 39) and may eventually be perpetuated and amplified by certain T cell types (such as TH17 cells). However, Th17 cells cannot initiate inflammatory reactions since their generation requires a previously inflammatory milieu (40). We did not fully block innate signaling in LM infection, since this signaling is complex and involves multiple mediators [reviewed in Ref. (41)]. However, the elimination of the MyD88 pathway reported to be the most important in LM infection (42) did not modify the pro-inflammatory burst. The strong inflammatory bursts of CD8 T cells after sterile immunizations with male, sheep red blood or allogeneic cells indicate that CD8 cognate interactions are also able to initiate inflammatory reactions. This feature may have an important role in a number of “sterile” responses to tissue antigens (such as in transplantation) or in responses to self-antigens or tumor antigens. Thus, while inflammation modulates cognate responses, CD8 cognate responses may also initiate local inflammatory reactions.

## Author Contributions

Hsueh-Cheng Sung and Sara Lemos performed most experiments; Patricia Ribeiro-Santos and Florence Vasseur did the cell sorting; Kateryna Kozyrytska, the CD4 studies, Agnès Legrand helped with the single-cell PCRs; César Evaristo trained Hsueh-Cheng Sung and contributed to the experiment shown in Figure 2A. Alain Charbit contributed to LM preparation, Benedita Rocha devised and supervised the study, and wrote the MS.

## Conflict of Interest Statement

The authors declare that the research was conducted in the absence of any commercial or financial relationships that could be construed as a potential conflict of interest.

## Supplementary Material

The Supplementary Material for this article can be found online at http://www.frontiersin.org/journal/10.3389/fimmu.2013.00452/abstract
