# Supplementary figures and images for: Cognate Antigen Stimulation Generates Potent CD8+ Inflammatory Effector T Cells
Source: Front Immunol. 2013 Dec 16;4:452. doi: 10.3389/fimmu.2013.00452 (PMC3863990; doi:10.3389/fimmu.2013.00452)

# Abs. SYBR Green

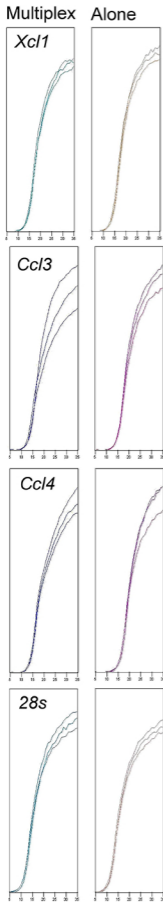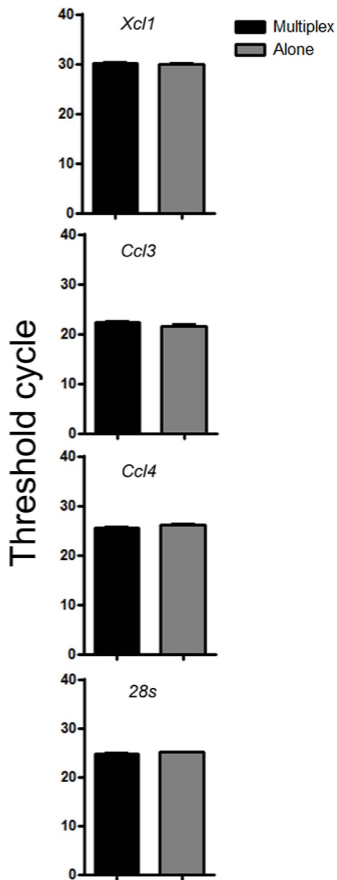

Supplement: Supplementary file 1 [file 68819_Rocha_Presentation1.PDF]

## A 1st PCR

Abs. SYBR Green

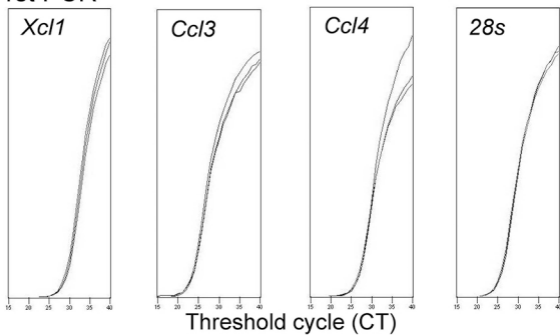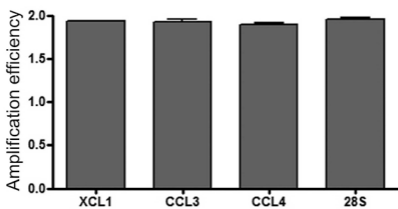

## B 2nd PCR

Abs. SYBR Green

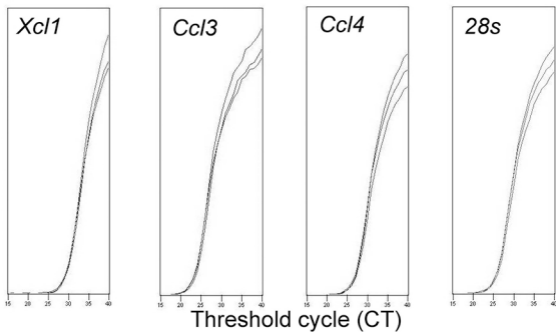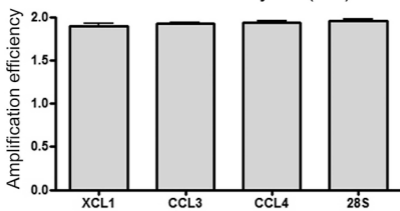

Supplement: Supplementary file 2 [file 68819_Rocha_Presentation2.PDF]
